# Identification and validation of immune and cuproptosis - related genes for diabetic nephropathy by WGCNA and machine learning

**DOI:** 10.3389/fimmu.2024.1332279

**Published:** 2024-02-08

**Authors:** Yubing Chen, Lijuan Liao, Baoju Wang, Zhan Wu

**Affiliations:** ^1^ Division of Hepatobiliary Surgery, The First Affiliated Hospital of Guangxi Medical University, Nanning, China; ^2^ Guangxi Key Laboratory of Immunology and Metabolism for Liver Disease, The First Affiliated Hospital of Guangxi Medical University, Nanning, China; ^3^ Department of Pathology, Xiangyang Central Hospital, Affiliated Hospital of Hubei University of Arts and Sciences, Xiangyang, China

**Keywords:** diabetic nephropathy, bioinformatic analysis, machine learning, WGCNA, biomarker

## Abstract

**Background:**

As the leading cause of chronic kidney disease, diabetic kidney disease (DKD) is an enormous burden for all healthcare systems around the world. However, its early diagnosis has no effective methods.

**Methods:**

First, gene expression data in GEO database were extracted, and the differential genes of diabetic tubulopathy were obtained. Immune-related genesets were generated by WGCNA and immune cell infiltration analyses. Then, differentially expressed immune-related cuproptosis genes (DEICGs) were derived by the intersection of differential genes and genes related to cuproptosis and immune. To investigate the functions of DEICGs, volcano plots and GO term enrichment analysis was performed. Machine learning and protein-protein interaction (PPI) network analysis helped to finally screen out hub genes. The diagnostic efficacy of them was evaluated by GSEA analysis, receiver operating characteristic (ROC) curve, single-cell RNA sequencing and the Nephroseq website. The expression of hub genes at the animal level by STZ -induced and db/db DKD mouse models was further verified.

**Results:**

Finally, three hub genes, including *FSTL1*, *CX3CR1* and *AGR2* that were up-regulated in both the test set GSE30122 and the validation set GSE30529, were screened. The areas under the curve (AUCs) of ROC curves of hub genes were 0.911, 0.935 and 0.922, respectively, and 0.946 when taking as a whole. Correlation analysis showed that the expression level of three hub genes demonstrated their negative relationship with GFR, while those of *FSTL1* displayed a positive correlation with the level of serum creatinine. GSEA was enriched in inflammatory and immune-related pathways. Single-nucleus RNA sequencing indicated the main distribution of *FSTL1* in podocyte and mesangial cells, the high expression of *CX3CR1* in leukocytes and the main localization of *AGR2* in the loop of Henle. In mouse models, all three hub genes were increased in both STZ-induced and db/db DKD models.

**Conclusion:**

Machine learning was combined with WGCNA, immune cell infiltration and PPI analyses to identify three hub genes associated with cuproptosis, immunity and diabetic nephropathy, which all have great potential as diagnostic markers for DKD and even predict disease progression.

## 1 Introduction

Diabetic kidney disease (DKD), the main complication of diabetes, mainly features hypertension, proteinuria and progressive decreases in kidney function. It is also a leading cause of chronic and even end-stage kidney diseases worldwide (1–3). DKD is expected to experience an increase of nearly 50% in morbidity in the next two decades, which results in an estimated 783 million patients. It is estimated that the 10-year cumulative mortality rate of DKD patients is up to 31.1% (4). Detecting DKD early is important to prevent its evolution into renal failure. Unfortunately, only abnormal proteinuria can be found due to the silence at the early stages of the disease, which makes its diagnosis difficult. Various studies have identified associations of DKD with markers of renal tubule injury such as *kidney injury molecule 1* (*KIM-1*), *neutrophil gelatinase-associated lipocalin* (*NGAL*) and *N-acetyl-β-D-glocosaminidase* (*NAG*), inflammatory markers like *tumor necrosis factor-α* (*TNF-α*) and *interleukin-1β* (*IL-1β*), as well as markers of oxidative stress like *8-hydroxy-2’-deoxyguanosine* (*8-OHdG*) (5). However, no available biomarkers can accurately diagnose or predict the progression of DKD.

Tubular injury in the progression of DKD has been recognized to play a significant role for the past few years and is named diabetic tubulopathy (6). Regarding mechanism, oxidative stress, hypoxia, albumin overload, inflammation, epithelial-mesenchymal transition (EMT), cellular senescence and nutrient-sensing pathways in tubule cells continue to occur during the development of diabetic nephropathy. They jointly promote the progression of tubule injury and interstitial fibrosis, which thus affects the prognosis of DKD patients (7).

Cuproptosis, a novel copper ion-dependent cell death regulatory process, has recently been recognized to be strongly correlated with mitochondrial respiration and the lipoic acid (LA) pathway (8). As an important micronutrient in the human body, copper is a key catalytic cofactor, which is vital in mitochondrial respiration, antioxidant defense, biocompound synthesis and other biological processes. Its concentration has been maintained in a low range. When accumulating abnormally, copper ions show serious cytotoxicity. However, the specific mechanisms that have evolved to do that are not fully understood.

Proximal tubules are the main site of renal reabsorption, in which a large number of mitochondria are distributed to produce energy (9). In the early phase of DKD, high glomerular filtration is accompanied by the increased reabsorption of proximal tubules. This causes a sharp increase in energy consumption and the exposure of proximal tubules to hypoxia, which leads to mitochondrial dysfunction. It was hypothesized that the process of diabetic renal tubular disease is accompanied by copper death, given the close relationship between cuproptosis and mitochondrial function. Recent studies have shown that all the stages of DKD are accompanied by immune infiltration, predominantly macrophage infiltration. In addition, the degree of immune infiltration has also been observed to have a close association with the progression of end-stage renal disease (ESRD) in DKD patients (10, 11). Therefore, the aim of this research was to investigate the associations of immune infiltration and cuproptosis with diabetic tubulopathy, and then construct and externally validate a novel DKD prediction model.

**Figure 1:**
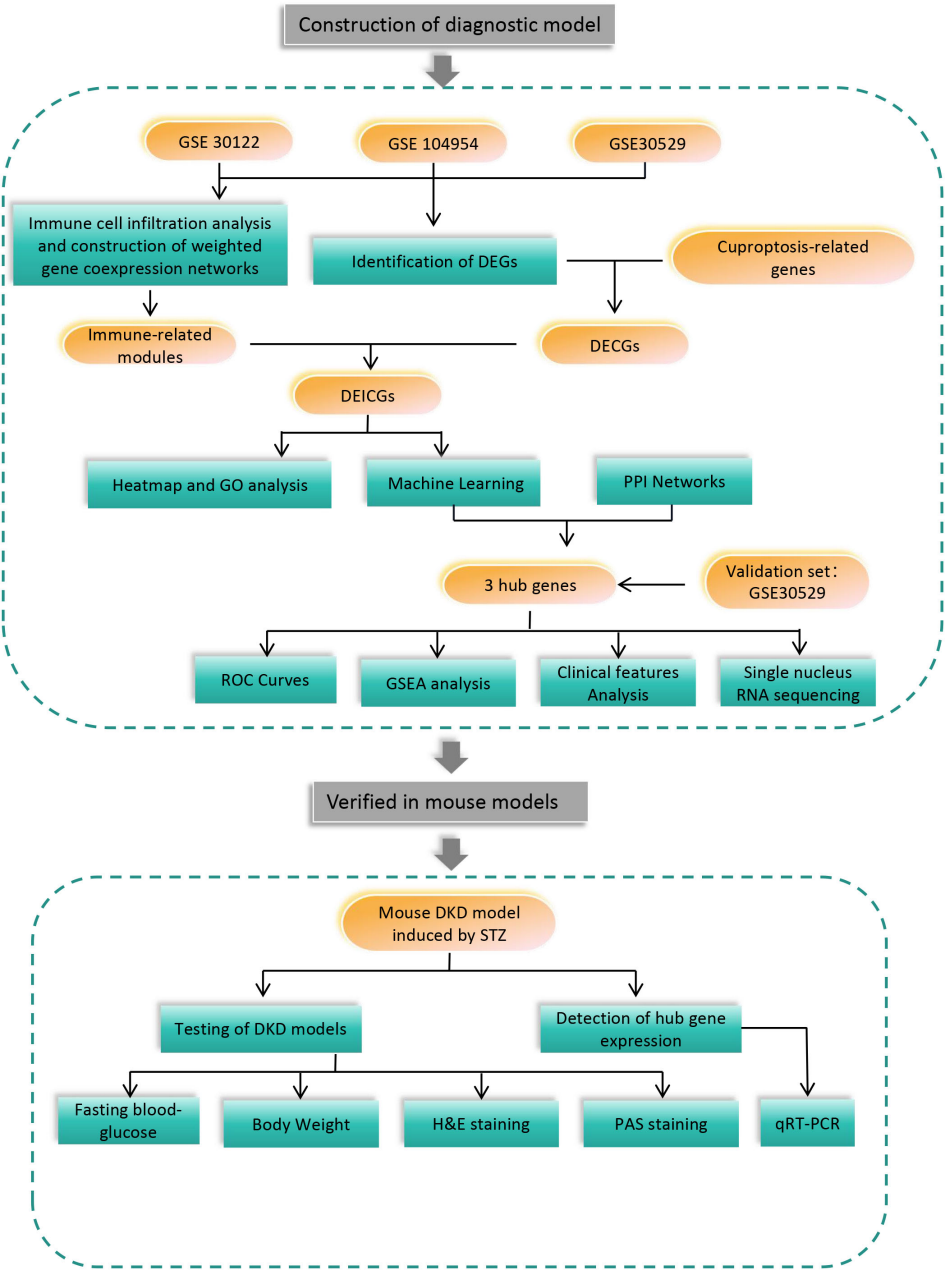
Flow chart for the study.

In this study, the diagnostic genes of DKD tubule injury were predicted by making a combination of the bioinformatics methods related to immune infiltration and cuproptosis. Additionally, the external data set was used as the verification set, and mouse models were also involved. The specific process of the experiment is shown in **Figure 1**.

## 2 Materials and methods

### 2.1 Data acquisition

The Gene Expression Omnibus (GEO) database was searched, and three data sets were obtained from renal tubules in diabetic nephropathy patients, namely gene set enrichment 30122 (GSE30122), GSE104954 and GSE30529, which were derived from the GPL571 and GPL22945 platforms, respectively. Their detailed information is shown in **Table 1**. A cuproptosis-related geneset was constructed by searching for “cuproptosis” in Gene Set Enrichment Analysis (GSEA) and GeneCards databases (https://www.gsea-msigdb.org)(https://www.genecards.org), respectively. After that, they were merged with key genes for cuproptosis mentioned in an article published in 2022 (8). The specific gene list is shown in **Supplementary Table 1**.

**Table 1:** Summary of the data sets utilized in this research and their features.

Dataset	Database	Platform	Sample	Tissue
GSE30122	GEO	GPL571	10 cases of DN and 24 cases of controls	Tubulointerstitium
GSE104954	GEO	GPL22945	7 cases of DN and 3 cases of controls	Tubulointerstitium
GSE30529	GEO	GPL571	10 cases of DN and 12 cases of controls	Tubulointerstitium

Three gene expression datasets from renal tubule samples from DKD patients were incorporated in our research, as detailed in the table.

### 2.2 Identification of differentially expressed genes

Firstly, difference analysis was conducted on the three GEO datasets respectively by the R package “limma”, and volcano maps were drawn with the help of the “ggplot” package. It was considered that P-value < 0.05 and |log2FC| >0.5 had statistical significance. A summary was made on the basis of the above three sets of differentially expressed genes(DEGs) and cuproptosis-related genes. A Venn diagram was plotted to obtain the collection of differential genes in diabetic nephropathy tubular tissues associated with copper death, which were named differentially expressed cuproptosis-related genes (DECGs).

### 2.3 Immune infiltration and weighted correlation network analysis

The cibersort platform (https://cibersortx.stanford.edu) helped us identify immune cell infiltration in diabetic nephropathy and control samples by matching their M22 dataset with the expression profile of this study. Weighted correlation network analysis (WGCNA) is a systems biology approach. It is used for describing patterns of gene associations between various samples by identifying high-covariance gene sets and candidate treatment targets or biomarker genes based on the interconnectivity of gene sets and the connection between gene sets and clinical features. Specifically, gene expression profiles were utilized to calculate the median absolute deviation (MAD) for each gene. The top 50% of genes with the smallest MAD were eliminated, and the “goodSamplesGenes” method of the R software package “WGCNA” was employed to remove outlier genes and samples. It was found that six modules, including dark orange, bisque4, darkgrey, orangered4, cyan and darkslateblue, exhibited the strongest correlation with immune cells. Therefore, they were selected for subsequent analysis. Afterwards, the genes in the DECGs gene set were intersected with the six key modules obtained by WGCNA. Intersection gene sets were named differentially expressed immune-related cuproptosis genes (DEICGs), and further research was carried out.

### 2.4 Gene ontology functional enrichment and GSEA analyses

The R software package “inSilicoMerging” was used for merging GSE104954 with GSE30122. Further, the batch effects between them were removed using the removeBatchEffect function of the R software package “limma”. The R package “ComplexHeatma” was adopted to generate heatmaps for visualization. For the functional enrichment analysis of gene sets, the gene ontology (GO) annotation of genes in the R software package “org.Hs.eg.db” was used for mapping genes into the background set. Enrichment analysis was performed using the clusterProfiler package. In the above process, the minimum and maximum gene sets were set to 5 and 5,000, respectively. It was considered that P value < 0.05 and false discovery rate (FDR) < 0.25 had statistical significance. In the follow-up study, the “clusterProfiler” package was also utilized to conduct GSEA enrichment analysis on the three finally selected hub genes.

### 2.5 Screening hub genes by machine learning and PPI networks

Least absolute shrinkage and selection operator (LASSO) regression is a constraint term model with L1 norm after the cost function of a linear regression model, which can be used for the variable screening and feature selection of high-dimensional data and model construction (12). The support vector machine-recursive feature elimination (SVM-RFE) algorithm is a method of machine learning that uses support vector machines as classifiers to identify the best core genes (13).

The protein interaction networks of seven proteins were retrieved from the STRING website (https://cn.string-db.org/) and imported into Cytoscape for visualization. With the aid of the cytohubba plug-in software in Cytoscape, eight kinds of algorithms such as boeeleneck, closeness, degree, density of maximum neighborhood component (DMNC), eccentricity, edge percolated component (EPC), maximal clique centrality (MCC) and newsun were obtained to forecast key genes. The top four genes in the results obtained by each algorithm were intersected. The intersection genes that overlapped in at least five algorithms were taken as a new gene set. The differential genes obtained by machine learning were further intersected. Finally, hub genes were obtained. The receiver operating characteristic (ROC) analysis of three hub genes was conducted by use of the “pROC” package and visualized by the “ggplot2” package.

### 2.6 Clinical analysis

The expression levels of three hub genes were compared using unpaired two-sample t-tests in the training cohort GSE30122 and the validation cohort GSE30529. Then, the correlations between the expression levels of hub genes and the serum creatinine and glomerular filtration rate (GFR) of clinical patients on Nephroseq(http://v5.nephroseq.org), a comprehensive information website related to kidney diseases, were searched.

### 2.7 Single nucleus RNA sequencing

The Kidney Integrative Transcriptomics (K.I.T.) database was invented in the laboratory of Ben Humphrey at Washington University (http://humphreyslab.com/SingleCell/) (14). Thanks to the database, the single-cell sequencing data of three hub genes in the renal tubular lesion samples of diabetic nephropathy were mined in this study, and the results were visualized.

### 2.8 DKD mouse models

The type 1 diabetes was induced by low-dose streptozocin (STZ) treatment in C57 male mice aged 8-10 weeks. All the mice received 12-week and 5-day feeding with a 60% high fat diet (HFD) (Guangdong Medical Animal Center, Guangzhou, China) and were given an intraperitoneal injection of STZ or citrate buffer after a 4-hour fast for 5 days before the start of feeding. Fasting blood glucose was measured one week after drug withdrawal. Mice with fasting blood glucose greater than 15mmol/L were considered to have successfully constructed a diabetes model. For the validation of diabetic nephropathy, the increase of urinary albumin level is an important indicator. Tubular atrophy, glomerular enlargement, thickened capsule and inflammatory cell infiltration in H&E staining and mesangial hyperplasia, basement membrane thickening and glomerular sclerosis with PAS staining can further verify the renal damage caused by hyperglycemia At the same time, we introduced 12-week-old male db/db mice from Shanghai Model Organisms Center, Inc. to model type 2 diabetes, with db/m mice as controls.

### 2.9 Hematoxylin-eosin and periodic acid-Schiff staining

Mouse kidneys were fixed with 4% paraformaldehyde immediately after sacrifice. After tissue processing and paraffin embedding, sections of 2 μm were cut for further staining. Hematoxylin-eosin (H&E) staining on kidney sections was performed according to standard H&E staining protocols, in which the staining time of hematoxylin and eosin was 18 minutes and 30 seconds, respectively. Periodic acid-Schiff (PAS) staining for glycogen was performed using the Solarbio kit (G1281).

### 2.10 RNA extraction from mouse kidney and quantitative real-time polymerase chain reaction

Ribonucleic acids (RNAs) were extracted by use of TRIzol [Invitrogen, Carlsbad, California (CA), the United States of America (USA)], followed by chloroform extraction. Subsequently, RNA precipitated in isopropanol and resuspended in nuclease-free double distilled water (ddH2O). Complementary deoxyribonucleic acids (cDNA) were synthesized using 1 μg corresponding RNAs (Takara Bio, Japan). Total cDNAs were diluted at a ratio of 1:3, and utilized for quantifying the three genome segments by SYBR Green qPCR (Vazyme,027E2220GB) with the following primer pairs: *FSTL1*: Forward: 5’-CTCCCACCTTCGCCTCTAAC-3’, Reverse: 3’-TTCTAGGTTCCTCCTCGCCG-5’, *GAPDH*: Forward:5’-GTTGGTTGGAGCAAACATCCC-3’, Reverse: 3’- TTAGGAGTGGGGGTGGCTTT-5’, *AGR2*: Forward: 5’-GTGGGAAGCCCAGATTTGCC-3’, Reverse: 3’-TAGTTTGGGCCGAGAGTCCT-5’, *CX3CR1*: Forward: 5’-CCATCTGCTCAGGACCTCAC-3’, Reverse: 3’-CACCAGACCGAACGTGAAGA-5’.The amplification process was completed on a 7500 fast real-time PCR System (Applied Biosystems, CA, USA).

### 2.11 Statistical analysis

Statistical Package for the Social Sciences (SPSS) software 23.0 (SPSS Inc., Chicago, USA) or GraphPad Prism 9 (GraphPad Software, CA, USA) was used to conduct statistical analysis. The expression levels of immune cells in diabetic nephropathy (DN) and control groups from the Cibersort website and those of hub genes in both groups were compared using unpaired t-tests. Statistical significance was set when p value was below 0.05.

## 3 Results

### 3.1 Identification of differential genes

**Figure 2:**
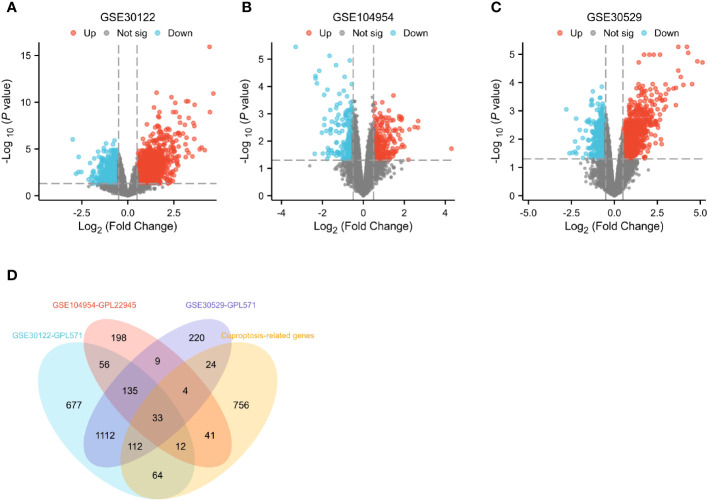
Identification of differential genes. **(A–C)** Differential gene expression on training cohort (GSE30122, GSE104954 and GSE30529). **(D)** Venn diagrams of differentially expressed genes.

Differential genes associated with renal tubulointerstitial damage in mice with diabetic nephropathy were searched for by downloading three gene sets, including GSE30122, GSE104954 and GSE30529, from the GEO database. The package “limma” was used for differential analysis to obtain 2,201, 488 and 1,649 diabetic nephropathy differential genes associated with tubulointerstitial damage in three datasets, respectively. (**Figures 2A–C**). The above gene sets were intersected with 1,046 cuproptosis-related gene sets collected by literature review and database mining. Then, a DECG gene set with 33 genes was obtained. (**Figure 2D**).

### 3.2 Immune infiltration analysis and construction of weighted gene co-expression networks

**Figure 3:**
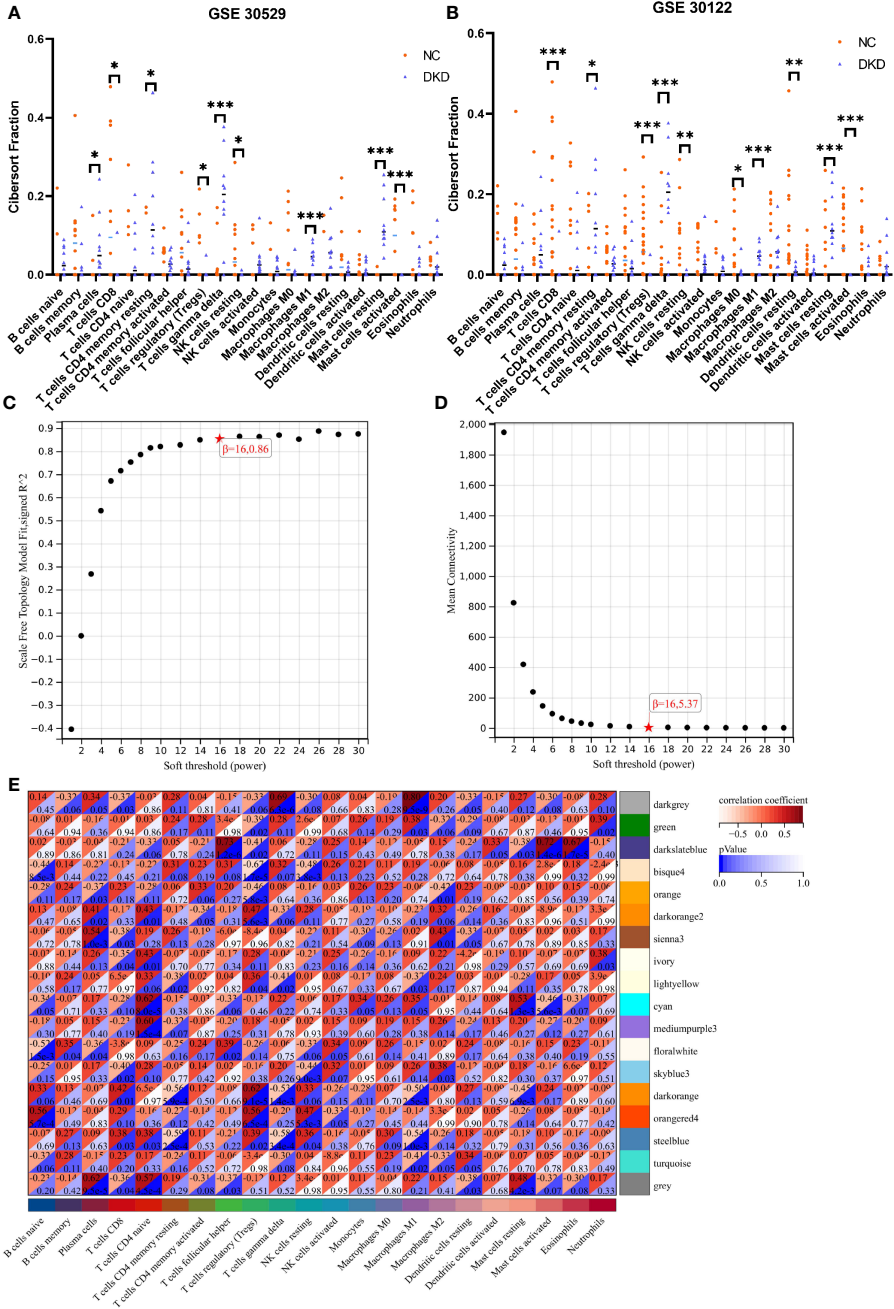
Immune infiltration analysis and construction of weighted gene co-expression networks. **(A, B)** Comparison of infiltration levels of immune cells in DKD group and control group inGSE30529 and GSE30122 database. **(C, D)**. The mean connectivity plot and scale-free topology plots, 16 was an appropriate soft-power. **(E)** 18 modules revealed by the WGCNA analysis. DKD, diabetic kidney disease; WGCNA, weighted gene co-expression network analysis. p values were showed as: *,p < 0.05; **,p < 0.01, ***,p <0.001.

Cibersort analysis suggested that the infiltration levels of nine kinds of immune cells in the GSE30529 database showed statistical differences in the tubulointerstitial damage of the diabetic nephropathy group. These immune cells included plasma cells, T cells cluster of differentiation 8 (CD8), T cells CD4 memory resting, T cells regulatory (Tregs), T cells gamma delta, natural killer (NK) cells resting, macrophages M1, and mast cells resting and activated. (**Figure 3A**). Meanwhile, 10 immune cell types were also shown to differ in infiltration level between DKD and control groups in the GSE 30122 dataset. These immune cell types contained T cells CD8, CD4 memory resting, Tregs, T cells gamma delta, NK cells resting, macrophages M0 and M1, dendritic cells resting, and mast cells resting and activated. (**Figure 3B**) In addition, two differentially distributed immune infiltrating cells were in the GSE104945 dataset, namely dendritic cells and mast resting. (**Supplementary Figure 1**).

The soft-threshold power, which was more than a scale-free topology fit index of 0.86 for each network, was 16. Thus, 16 was selected as the soft power threshold for the construction of WGCNA. (**Figure 3C**). In the meantime, the network connectivity under different soft thresholds is shown in **Figure 3D**. Subsequently, 18 modules were presented in the analysis process. Among them, six modules, including dark orange, bisque4, darkgrey, orangered4, cyan and darkslateblue, demonstrated the strongest positive correlation with the infiltration level of immune cells and were taken into consideration for further investigation (**Figure 3E**).

### 3.3 Identification and GO enrichment of DEICGs

**Figure 4:**
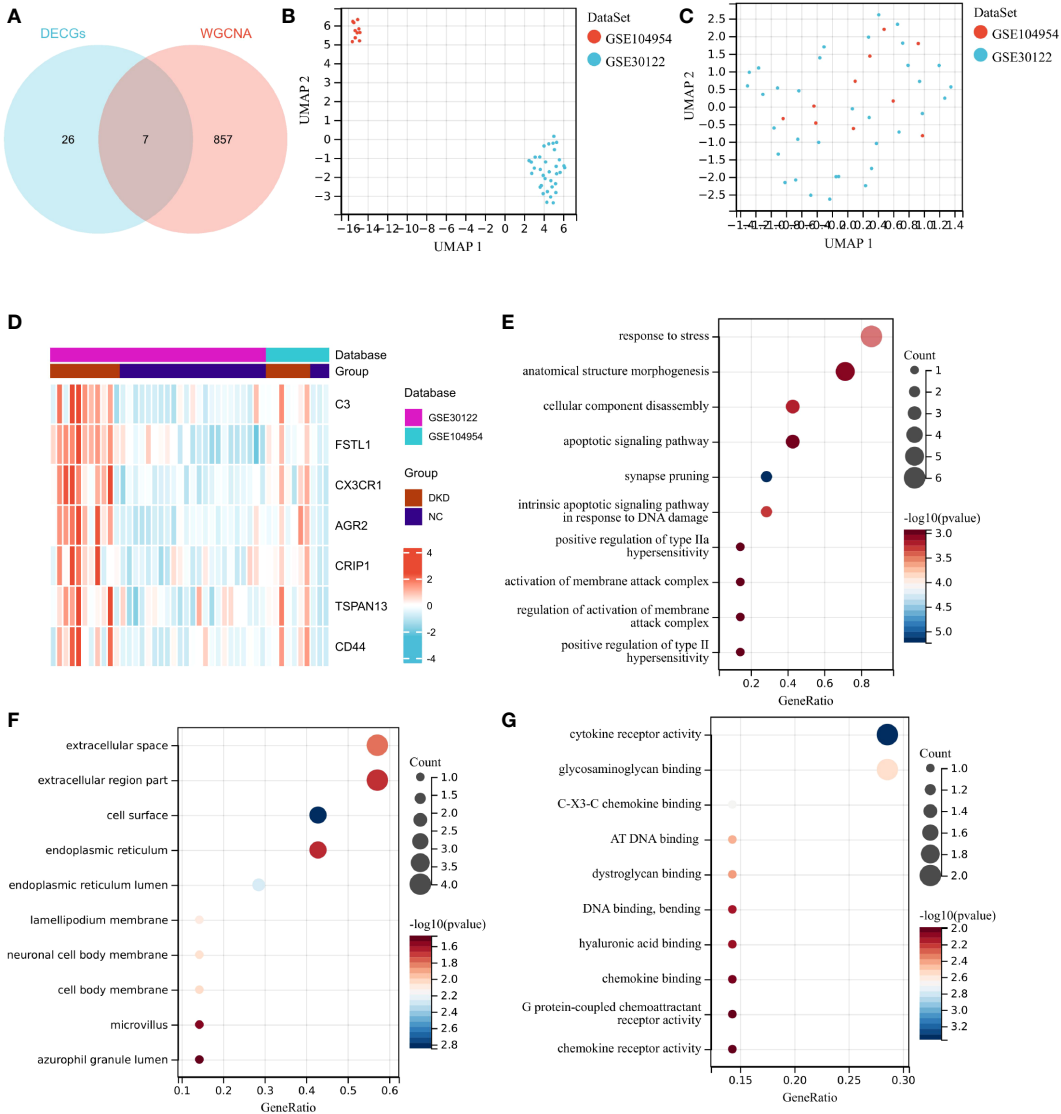
Identification of DEICGs and GO enrichment of them. **(A)** The immune-related genes screened by WGCNA analysis were combined with DECGs to draw the Venn diagrams, and thus DEICGs was obtained. **(B, C)** Sample distribution of GSE104954 and GSE30122 data sets before and after batch effect correction. **(D)** Distribution of DEICGs in GSE104954 and GSE30122 datasets. **(E–G)** Gene functions were categorized based on BP, MF, and CC by GO enrichment analysis. DECGs, differentially expressed cuproptosis-related genes; DEICGs, differentially expressed immune-related cuproptosis genes; BP, Biological Process; CC, Cell Component; MF, Molecular Function).

DEICGs were further harvested by intersecting the six correlation modules obtained from WGCNA with the DECGs obtained from the previous analysis. (**Figure 4A**). To increase the representativeness of the subsequent results, the GSE104954 dataset was combined with the GSE30122, and the batch effect was removed. (**Figures 4B, C**). Increased messenger RNA (mRNA) levels of DEICGs were observed in the heatmaps of two datasets. (**Figure 4D**). The GO enrichment analysis results demonstrated that DEICGs mainly got involved in biological processes like response to stress and anatomical structure morphogenesis, were distributed in cellular components like extracellular space, and played a role in cytokine receptor activity and other molecular functions (**Figures 4E–G**).

### 3.4 Screening of hub genes by machine learning and PPI networks

**Figure 5:**
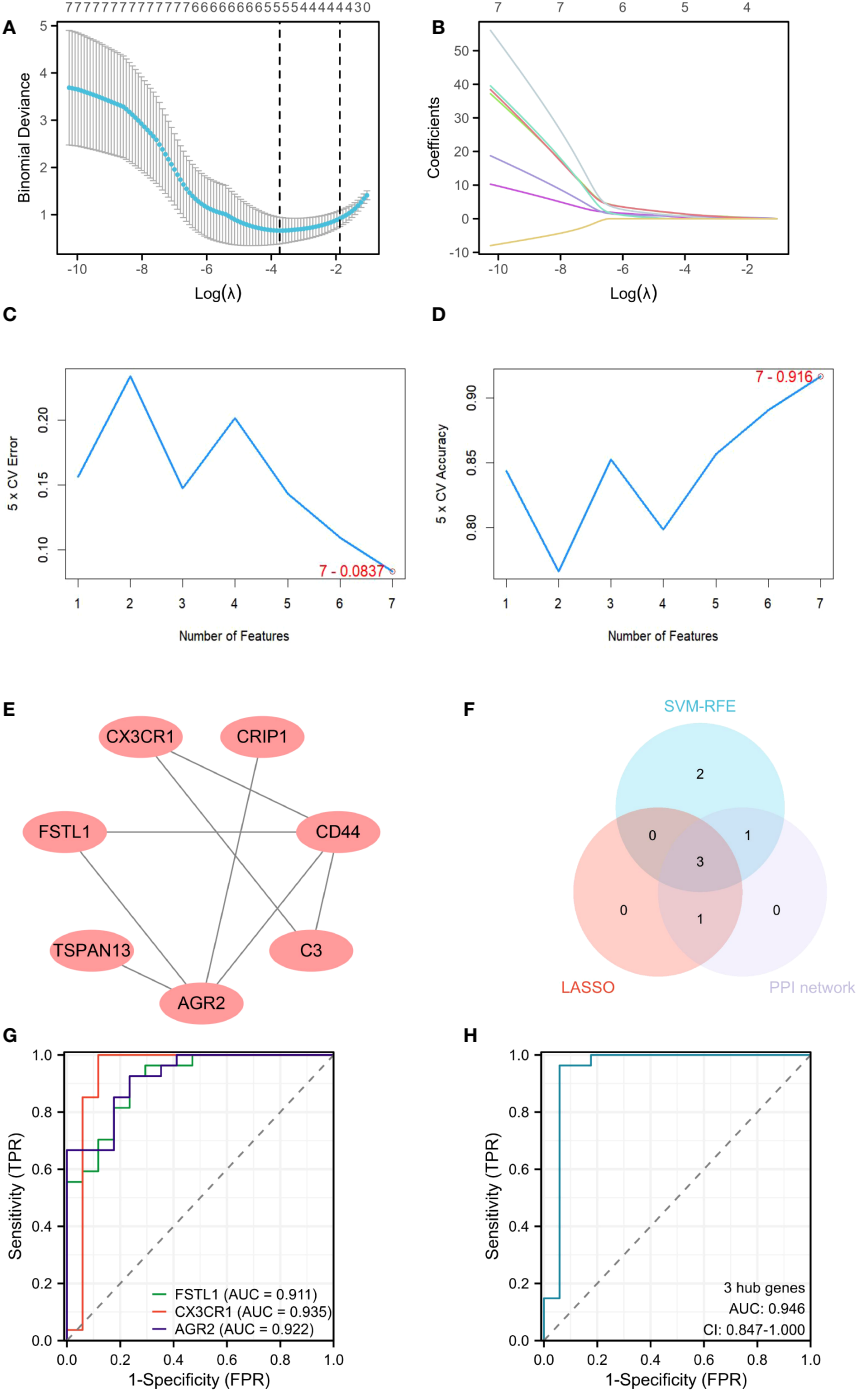
Screening hub genes by machine learning. **(A, B)** Cross-validation curves and regression coefficient path diagram in LASSO logistic regression algorithm. **(C, D)** The curve of change in the predicted true and error value of each gene in SVM-RFE algorithm. **(E)** Plot the PPI interaction network for DEICGs. **(F)** Venn diagram demonstrates the intersection of the above three analyses. **(G)** ROC curve analysis for 3 hub genes. **(H)** ROC analysis of prediction model composed of 3 hub genes. LASSO, least absolute shrinkage and selection operator; SVM-RFE, support vector machine-recursive feature elimination; PPI, protein-protein interaction; ROC, receiver operating characteristic.

A machine learning algorithm model was further built to extract key genes from DEICGs that have more diagnostic significance for diabetic nephropathy. We first tested the machine learning algorithm on the dataset merged with GSE30122 and GSE104954. Through the LASSO regression algorithm, a signature of five genes was obtained. (**Figures 5A, B**). As for SVM-RFE, seven genes experienced the lowest classifier error and highest classifier accuracy (minimal error = 0.0837, maximal accuracy = 0.916) (**Figures 5C, D**). We then tested the above analysis with GSE30122, and five and seven genes were validated in LASSO regression algorithm and SVM-RFE analyses, respectively. The overlapping part of the test and validation results were selected to represent the final results of LASSO regression algorithm and SVM-RFE analyze to participate in the subsequent produce the final hub genes. Meanwhile, five genes were predicted from the PPI network by using the CytoHubba plug-in software in Cytoscape (**Figure 5E**). By intersecting the above three gene sets through the Venn diagram, three hub genes were obtained at last (**Figure 5F**). Among them, *FSTL1* has been proved to promote the progression of focal segmental glomerular sclerosis(FSGS), membranous nephropathy (MN), immunoglobulin A (IgA) nephropathy (IgAN), and other chronic kidney diseases. *CX3CR1* has been reported to regulate immune responses such as inflammation, cell adhesion, and chemotaxis, and therefore plays a crucial role in the progression of kidney diseases such as IgA nephritis, nephrotoxic nephritis, and renal candidiasis. The role of *AGR2* in DKD disease progression has been predicted but not confirmed by experimental data. Combined with the above information, we believe that the three hub genes have great potential as markers of progression of diabetic tubulopathy. In further evaluation, the ROC curve showed high AUC levels for *FSTL1*, *CX3CR1* and *AGR2*, which were 0.911, 0.935 and 0.922, respectively. The cut-off values of *FSTL1*,*CX3CR1* and *AGR2* genes were 4.636, 3.425 and 3.101, respectively. The sensitivity and specificity were 0.926 and 0.765 for *FSTL1*, 1 and 0.882 for *CX3CR1* and those of *AGR2* is 0.926 and 0.765 (**Figure 5G**). The above data indicated the three genes’ high diagnostic accuracy. When taken as a whole, the AUC of the ROC curve of the disease prediction model composed of the three hub genes was 0.946, the cut-off value was 0.633 and the sensitivity and specificity were 0.963 and 0.941 respectively (**Figure 5H**).

### 3.5 Expression of hub genes and validation of external datasets

**Figure 6:**
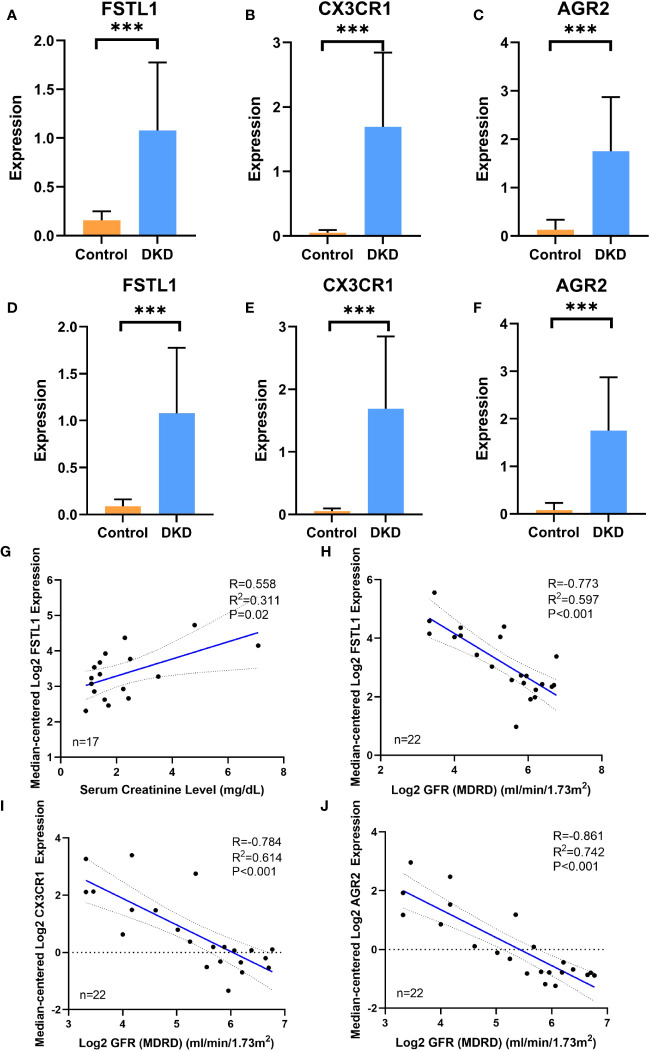
The expression of hub genes in disease database and its correlation with clinical features **(A–C)**. The expression level of FSTL1, CX3CR1 and AGR2 in DN group and control group in training cohort GSE30122. **(D–F)** The expression level of FSTL1, CX3CR1 and AGR2 in DKD group and control group in validation cohort GSE30529. **(G, H)** Correlation analysis of FSTL1 expression with serum creatinine and GFR level. **(I)** Correlation analysis of CX3CR1 expression with GFR level. **(J)** Correlation analysis of AGR2 expression with GFR level. GFR, glomerular filtration rate. p values were showed as: ***,p <0.001.

Both the training set GSE30122 and the validation set GSE30529 from the GEO database showed a consistent increase in the mRNA levels of three hub genes, including *FSTL1*, *CX3CR1* and *AGR2*, in the DKD group. (**Figures 6A–F**). When it comes to clinical patients, correlation analysis showed that the expression level of *FSTL1* had a positive correlation with the level of serum creatinine (p=0.02, R^2^ = 0.311) and a negative correlation with GFR(p<0.001, R^2^ = 0.597) (**Figures 6G, H**). The expression levels of *CX3CR1* and *AGR2* also displayed a negative correlation with GFR (p1<0.001,p2<0.001,R1^2^ = 0.614,R2^2^ = 0.742) (**Figures 6I, J**).

### 3.6 GSEA analysis

**Figure 7:**
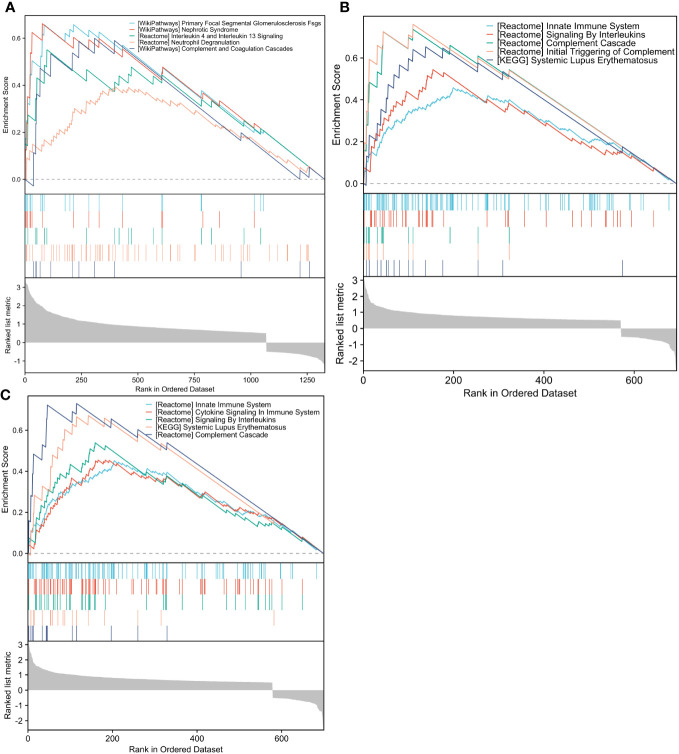
GSEA analysis of hub genes. **(A–C)**. The differential genes with high and low FSTL1, CX3CR1 and AGR2 expression in GSE30122 database were enriched by ESEA analysis. GSEA, Gene Set Enrichment Analysis.

GSEA analysis revealed that the *FSTL1* high-expression group was mostly concentrated in pathways like primary focal segmental glomerulosclerosis (FSGS), nephrotic syndrome, and IL-4 and IL-13 signaling and neutrophil degranulation (**Figure 7A**). The *CX3CR1* high-expression group was highly enriched for the innate immune system, signaling by ILs and the complement cascade (**Figure 7B**). Reactomes like the innate immune system, cytokine signaling in the immune system and signaling by ILs were all associated with elevated *AGR2* levels. (**Figure 7C**).

### 3.7 Single Nucleus RNA sequencing of hub genes

**Figure 8:**
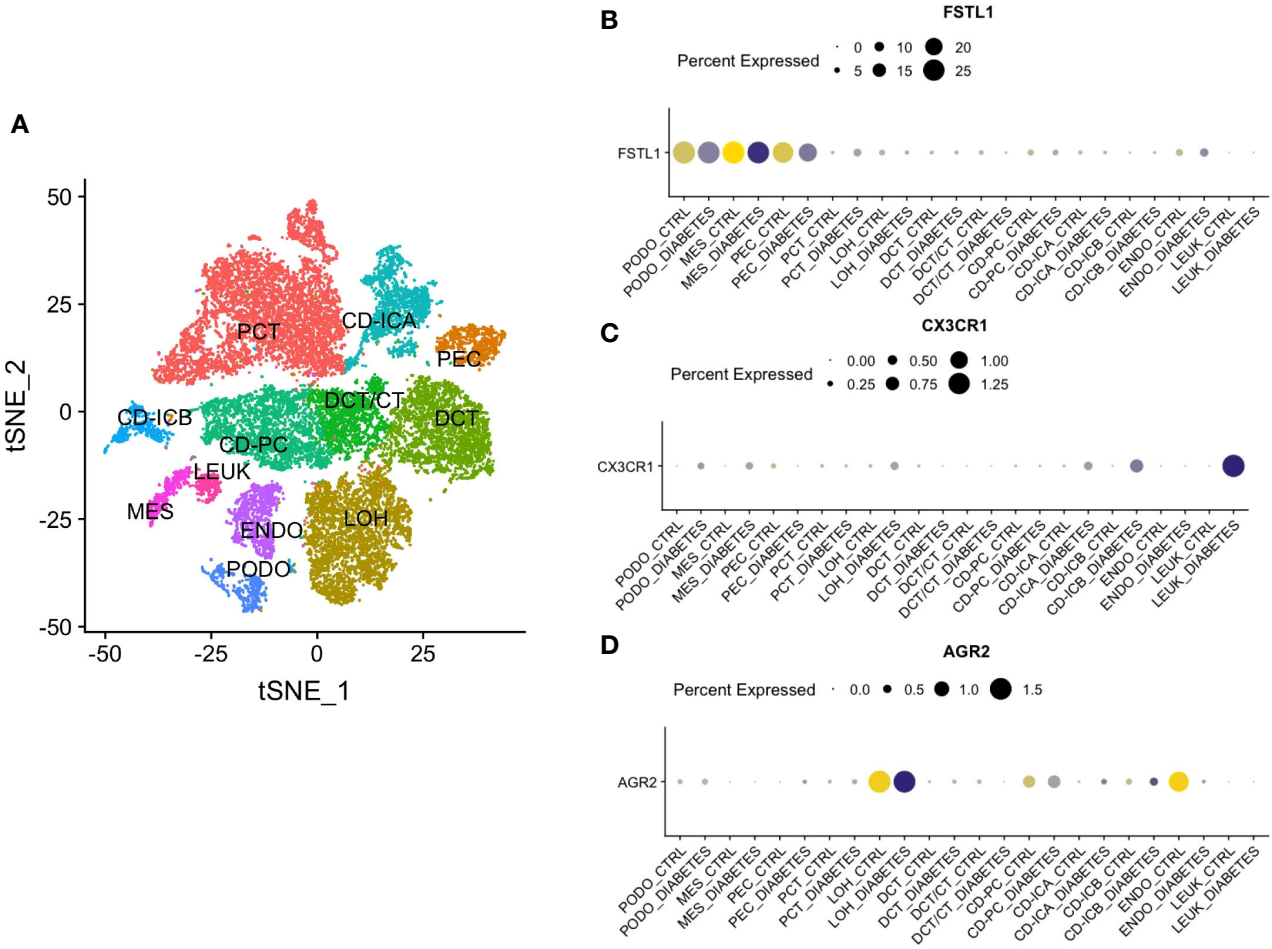
Single Nucleus RNA Sequencing of hub genes. **(A)** The division of 12 cell types. **(B)** The distribution of FSTL1. **(C)** The distribution of CX3CR1. **(D)** The distribution of AGR2. CD, collecting duct. ICA, Type A intercalated cells. ICB, Type B intercalated cells. PEC, parietal epithelial cells. PC, principal cell. DCT, distal convoluted tubule. CT, connecting tubule. LOH, loop of Henle. PODO, podocyte. ENDO, endothelium. MES, mesangial cell. LEUK, leukocyte.

Single-nucleus RNA sequencing was performed to identify how the three hub genes were distributed in 12 cell populations (**Figure 8A**). Among them, *FSTL*1 was mainly distributed in podocyte and mesangial cells (**Figure 8B**), *CX3CR1* was highly expressed in leukocytes (**Figure 8C**) and *AGR2* was mostly localized in the loop of Henle (**Figure 8D**).

### 3.8 Expression level of hub genes in the kidneys of DKD mice

**Figure 9:**
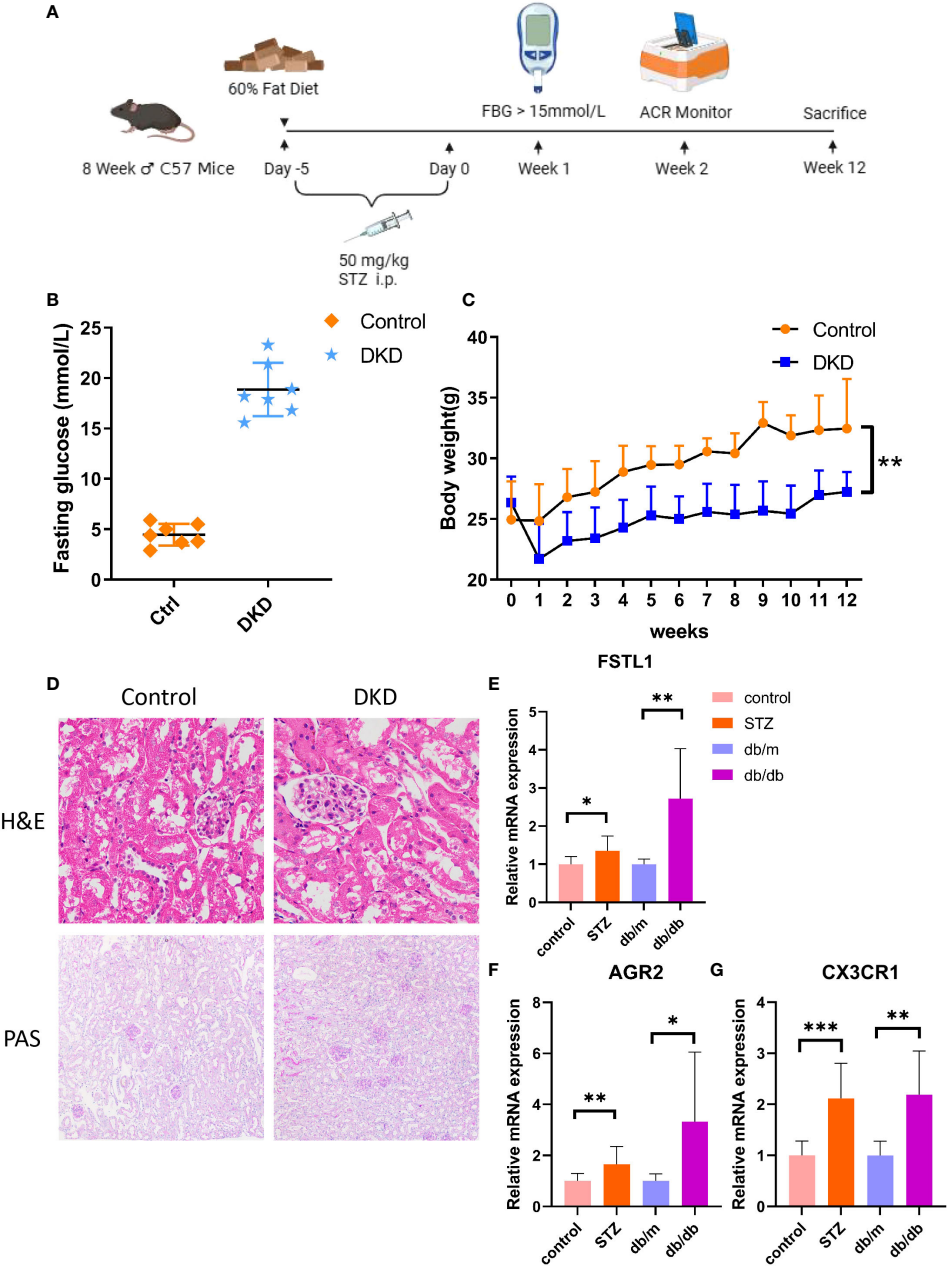
The expression level of hub genes in kidney of DKD mice. **(A)** Schematic diagram of STZ-induced mouse DKD models. **(B)** Fasting glucose in both control and STZ-induced DKD mice. **(C)** Body weight in both control and STZ-induced DKD mice. **(D)** The H&E and PAS staining in control and STZ-induced DKD mice. **(E–G)**. mRNA expression of FSTL1, AGR2 and CX3CR1 by RT-qPCR. p values were showed as: *,p < 0.05; **,p < 0.01, ***,p < 0.001. STZ, streptozocin; H&E, hematoxylin-eosin; PAS, schiff periodic acid shiff.

To assess the diagnostic value of hub genes at the mouse level, a mouse model of type 1 diabetes with STZ was constructed, and the specific methods are described in **Figure 9A**. One week after the injection, the fasting blood glucose of the DKD group was all greater than 15mmol/L, which indicated the successful construction of the diabetes model. (**Figure 9B**). After the mice were weighed weekly for 12 weeks, it was found that the DKD group was lighter than the control one, which is consistent with the clinical manifestations of diabetes (**Figure 9C**). H&E staining indicated that the DKD group was characterized by the hypertrophy of glomerular capillary loops, glomerular enlargement and the vacuolar degeneration of renal tubular epithelial cells (**Figure 9D**). PAS staining demonstrated mesangial hyperplasia, basement membrane thickening, glomerular sclerosis and other additional abnormalities in the DKD group (**Figure 9D**). The quantitative real-time polymerase chain reaction (RT-qPCR) results of the mouse renal cortex sample suggested that the expression levels of all hub genes showed an increase in both STZ treatment group and db/db group. (**Figures 9E–G**).

## 4 Discussion

Proximal tubular injury associated with DKD, a microvascular complication with the highest mortality rate in diabetes, has always been a vital focus of investigation on the pathogenesis of the disease (15). It has been shown that oxidative stress, hypoxia, albumin overload, inflammation, EMT, cellular senescence and dysfunctional nutrient-sensing pathways all play a vital role in the progression of diabetic tubulopathy (7). However, it remains difficult to diagnose DKD early due to the complexity of the disease, and effective biomarkers are insufficient to diagnose the disease and predict its prognosis. It is known that the progression of diabetic tubulopathy is closely related to immune infiltration and mitochondrial dysfunction (16).

In this study, a series of differentially expressed genes were obtained in DKD renal tubules by mining the GEO database. They were intersected with genes related to cuproptosis and immunity to get the DEICGs gene set. Machine learning algorithms, which process large amounts of biological data to group them and build models, are widely used for predicting disease markers and treatment targets (17). During the analysis process, genes were further screened with the help of machine learning, the WGCNA algorithm and PPI protein interaction analysis. Eventually, three hub genes were found, namely *FSTL1*, *CX3CR1* and *AGR2*. The ROC curve mapping of hub genes and the correlation analysis of the gene expression levels, serum creatinine and GFR of clinical patients further suggested their significant diagnostic and prognostic efficacy. To explore the possible mechanism of hub genes, GO and GSEA pathway enrichment analyses were also conducted on them. It was found that inflammatory and immune-related pathways like IL-4 and IL-13 signaling and the innate immune system occurred frequently, which may be crucial for the progression of DKD. After the validation of the aforementioned hug genes in the external dataset GSE30529, the higher levels of hub genes in STZ-induced type 1 and db/db type 2 diabetic mouse models were further demonstrated, respectively.

*FSTL1* is a secreted glycoprotein that is important in various physiological processes, such as angiogenesis, immune response regulation, and cell proliferation and differentiation. In human kidneys, single-cell transcriptomic data suggest that *FSTL1* is only distributed in interstitial fibroblasts and myofibroblasts (18). By activating nuclear factor-kappa B (NF-κB) and wingless (Wnt)/β-catenin pathways, *FSTL1* has been found to promote the progression of different forms of CKD, including focal segmental glomerular sclerosis(FSGS), membranous nephropathy (MN), immunoglobulin A (IgA) nephropathy (IgAN), etc. (19, 20). In the present study, the expression level of *FSTL1* in the renal tubules of DKD individuals was increased in both test and validation sets and displayed a positive correlation with the serum creatinine level of patients and a negative correlation with GFR levels. The above results were further validated in both type 2 and 1 diabetic mouse models. ROC analysis indicated that the AUC area of *FSTL1* in diabetic tubulopathy was 0.911, which indicated its strong diagnostic efficacy. The association between *FSTL1* and DKD has not been reported previously. However, it has been reported that *FSTL1* influences the progression of fibrosis in diabetic retinopathy, another type of diabetic microangiopathy, through the extracellular matrix (ECM) receptor pathway (21). GSEA strongly implicated that immune-related pathways such as IL-4 and IL-13 signaling, neutrophil degranulation and complement and coagulation cascades pathways are significantly affected by *FSTL1* in DKD.

*CX3CR1* belongs to the G-protein coupling receptor (GPCR) superfamily and is a cell surface receptor mostly expressed in T lymphocytes, monocytes and NK and mast cells (22–24). Studies suggest that *CX3CR1* regulates many aspects of the immune response, such as inflammation, cell adhesion and chemotaxis. *CX3CR1* is crucial in the progression of IgA nephritis, nephrotoxic nephritis, renal candidiasis and other renal diseases (25). Few relevant studies have focused on *CX3CR1* in DKD. It was only noted that *CX3CR1* was up-regulated in DKD many years ago. In the current study, *CX3CR1* was observed to be up-regulated in the renal tissues of DKD patients and have a diagnostic accuracy value (AUC = 0.935). The expression levels of *CX3CR1* were negatively correlated with GFR levels in DKD patients. The related mechanism mainly involved the innate immune system, signaling by interleukins and complement cascade pathways.

*AGR2* was screened from MCF7, an estrogen receptor positive breast cancer cell line, and its transcription is responsive to estrogen at the molecular level (26). *AGR2* has been implicated in inflammatory bowel disease and cancer progression (27, 28), and mechanically, promotes angiogenesis and increases the invasiveness of tumor cells (29). Previous bioinformatics studies predicted the efficacy of the *AGR2* gene in the detection of diabetic renal tubular disease but failed to verify their conclusions in disease samples (30). In this study, it was noticed that *AGR2* was up-regulated in the tubule tissues of DKD individuals and higher expression levels of *AGR2* were often associated with lower GER levels, which signified a close association between *AGR2* and diabetic tubulopathy progression. The ROC curve showed that *AGR2* had a high AUC level of 0.992, which indicated its high diagnostic accuracy. In addition, the mechanism of *AGR2* getting involved in DKD is mostly ascribed to the innate immune system, cytokine signaling in the immune system and signaling by interleukins.

Common mechanisms discovered in DKD include metabolic, hemodynamic, inflammatory and fibrotic mechanisms, each of which provides potential therapeutic targets (31–33). A plethora of studies have demonstrated the functional role of inflammation in DKD, which can be triggered by the interaction of metabolic defects, secondary injury mediators (like angiotensin II and aldosterone) and pathological consequences of kidney cell injury or dysfunction (such as albuminuria, loss of Klotho production, lipid accumulation and lipotoxicity) (34, 35). At this point, the infiltration of massive immune cells occurs. The enrichment analysis of hub genes found that they affect the progression of DKD mainly through immune and inflammatory pathways, which corresponds to the pathophysiological process of DKD and indicates their diagnostic efficacy.

In this study, three hub genes in diabetic tubulopathy, which are closely related to cuproptosis and immunity and have high diagnostic efficacy, were innovatively identified. Some limitations are worth noting. First, the specific mechanism of hub genes in DKD needs to be further verified, which is also the focus of our follow-up work. Moreover, the expression level and predictive performance of hub gene in other renal diseases that are often differentiated from diabetic nephropathy, such as hypertensive nephropathy, IgA nephropathy and membranous nephropathy, should also be considered, which is crucial for the subsequent application of the prediction model composed of these three hub genes, although their diagnostic efficacy of this model for diabetic kidney disease has been fully verified. As a result, it is necessary to test the expression of hub genes in other chronic kidney diseases in future work.

## 5 Conclusion

In this study, immune infiltration analysis was combined with WGCNA analysis, machine learning and PPI network analysis to obtain three valuable hub genes: *FSTL1*, *CX3CR1* and *AGR2*. Then, the valuable clinical efficacy of these hub genes was further demonstrated, and their possible mechanisms were predicted with the help of GSEA analysis, a ROC curve, single-cell RNA sequencing and some online clinical databases. Finally, a DKD model of C57 mice was constructed, and the expression of animal-level hub genes was verified. *FSTL1*, *CX3CR1* and *AGR2* all have great potential as diagnostic markers for DKD and even predict disease progression.

## Data availability statement

The original contributions presented in the study are included in the article/**Supplementary Material**. Further inquiries can be directed to the corresponding authors.

## Ethics statement

The animal study was approved by Animal Care and Use Committee of Guangxi Medical University. The study was conducted in accordance with the local legislation and institutional requirements.

## Author contributions

YC: Conceptualization, Formal Analysis, Investigation, Visualization, Writing – original draft. LL: Writing – original draft, Investigation, Validation. BW: Writing – review & editing, Data curation, Methodology, Software, Supervision. ZW: Funding acquisition, Project administration, Resources, Writing – review & editing.

## Supplementary material

Supplementary Figure 1 Comparison of infiltration levels of immune cells in DKD group and control group in GSE104954.

Supplementary Figure 2 Screening hub genes by machine learning in GSE30122. **(A, B)** Cross-validation curves and regression coefficient path diagram in LASSO logistic regression algorithm; **(C, D)** The curve of change in the predicted true and error value of each gene in SVM-RFE algorithm.

Supplementary Table 1 List of genes related to cuproptosis.
