# Supplementary figures and images for: Identification and validation of immune and cuproptosis - related genes for diabetic nephropathy by WGCNA and machine learning
Source: Front Immunol. 2024 Feb 8;15:1332279. doi: 10.3389/fimmu.2024.1332279 (PMC10881670; doi:10.3389/fimmu.2024.1332279)

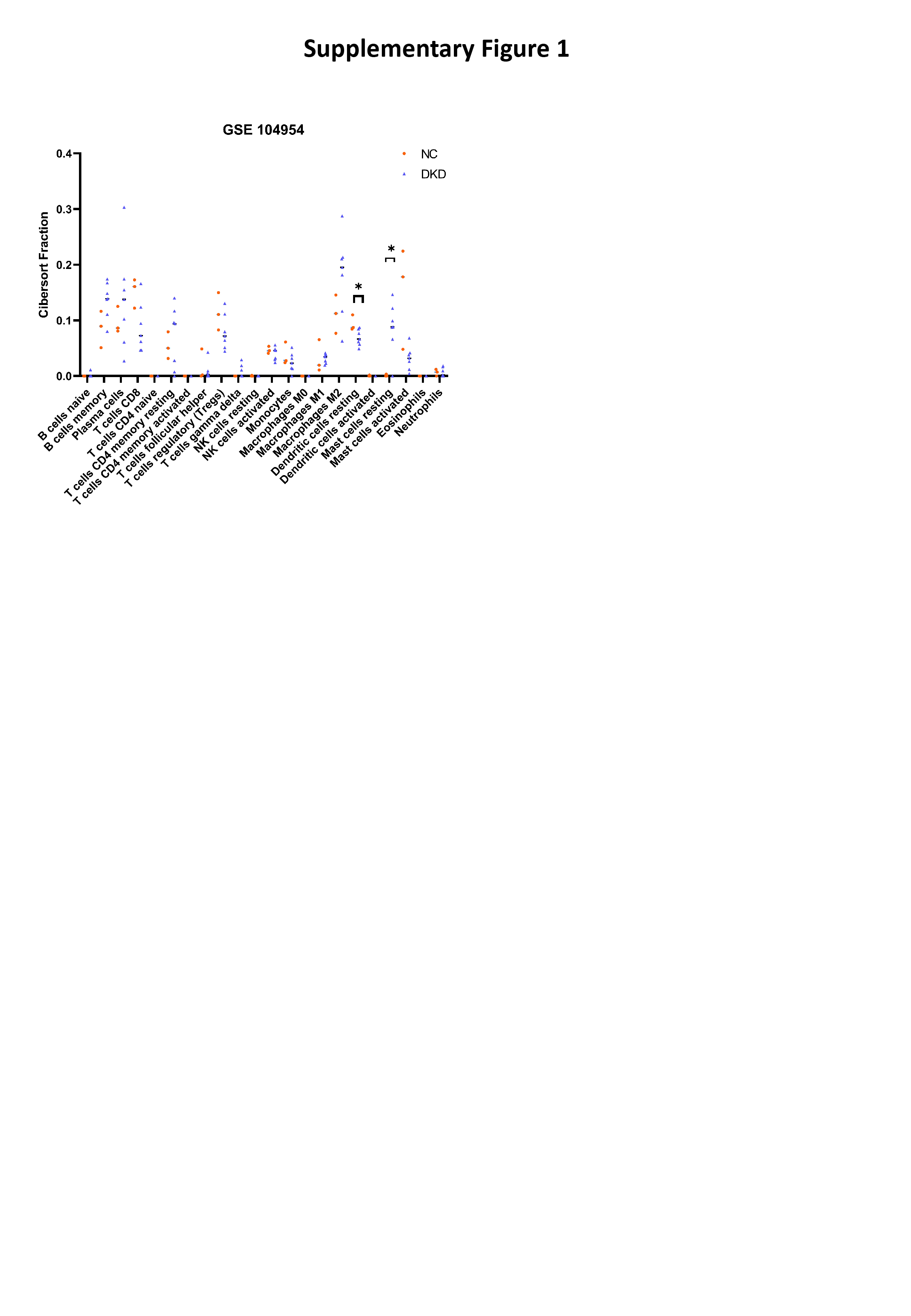

Supplement: Supplementary Figure 1 — Comparison of infiltration levels of immune cells in DKD group and control group in GSE104954. [file Image_1.tif]

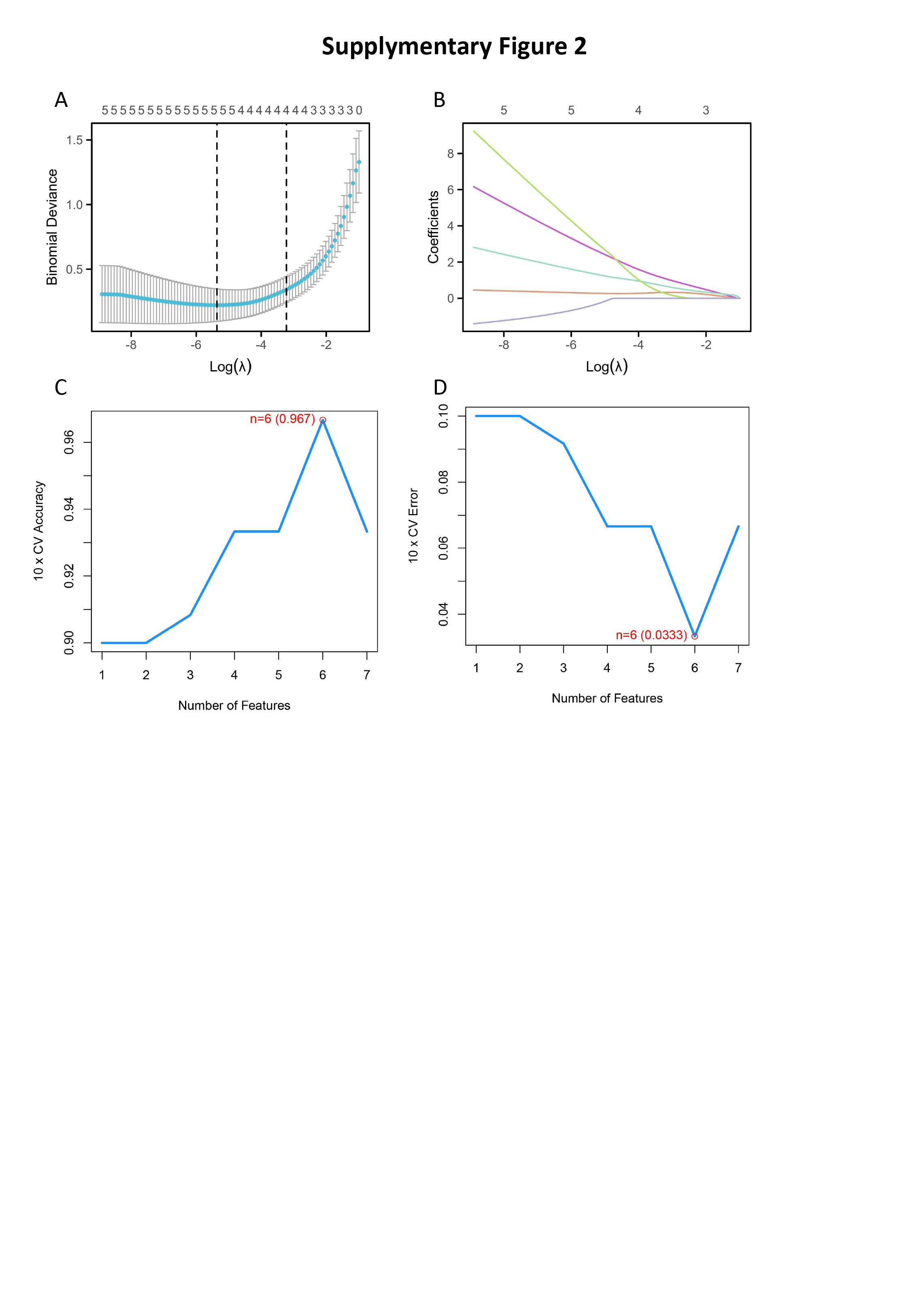

Supplement: Supplementary Figure 2 — Screening hub genes by machine learning in GSE30122. (A, B) Cross-validation curves and regression coefficient path diagram in LASSO logistic regression algorithm; (C, D) The curve of change in the predicted true and error value of each gene in SVM-RFE algorithm. [file Image_2.tif]
